# Phytoremediation: A Promising Approach for Revegetation of Heavy Metal-Polluted Land

**DOI:** 10.3389/fpls.2020.00359

**Published:** 2020-04-30

**Authors:** An Yan, Yamin Wang, Swee Ngin Tan, Mohamed Lokman Mohd Yusof, Subhadip Ghosh, Zhong Chen

**Affiliations:** ^1^Natural Sciences and Science Education, National Institute of Education, Nanyang Technological University, Singapore, Singapore; ^2^Centre for Urban Greenery and Ecology, National Parks Board, Singapore, Singapore; ^3^School of Environmental and Rural Science, University of New England, Armidale, NSW, Australia; ^4^M Grass International Institute of Smart Urban Greenology, Singapore, Singapore

**Keywords:** phytoremediation, heavy metal, uptake, detoxification, genetic engineering, chelate

## Abstract

Heavy metal accumulation in soil has been rapidly increased due to various natural processes and anthropogenic (industrial) activities. As heavy metals are non-biodegradable, they persist in the environment, have potential to enter the food chain through crop plants, and eventually may accumulate in the human body through biomagnification. Owing to their toxic nature, heavy metal contamination has posed a serious threat to human health and the ecosystem. Therefore, remediation of land contamination is of paramount importance. Phytoremediation is an eco-friendly approach that could be a successful mitigation measure to revegetate heavy metal-polluted soil in a cost-effective way. To improve the efficiency of phytoremediation, a better understanding of the mechanisms underlying heavy metal accumulation and tolerance in plant is indispensable. In this review, we describe the mechanisms of how heavy metals are taken up, translocated, and detoxified in plants. We focus on the strategies applied to improve the efficiency of phytostabilization and phytoextraction, including the application of genetic engineering, microbe-assisted and chelate-assisted approaches.

## Introduction

With the development of industrialization and urbanization, the abundance of heavy metals in the environment has increased enormously during the past decades, which raised significant concerns throughout the world (Suman et al., 2018; Ashraf et al., 2019). Heavy metals are a group of metallic chemical elements that have relatively high densities, atomic weights, and atomic numbers. The common heavy metals/metalloids include cadmium (Cd), mercury (Hg), lead (Pb), arsenic (As), zinc (Zn), copper (Cu), nickel (Ni), and chromium (Cr). These heavy metals/metalloids originate from either natural or anthropogenic sources such as produced water generated in oil and gas industries (Neff et al., 2011; Pichtel, 2016), use of phosphate fertilizers in agriculture (Hamzah et al., 2016; Rafique and Tariq, 2016), sewage sludge (Farahat and Linderholm, 2015), metal mining and smelting (Chen et al., 2016), pesticide application (Iqbal et al., 2016), electroplating, and fossil fuel burning (Muradoglu et al., 2015).

Heavy metals are non-degradable by any biological or physical process and are persistent in the soil for a long period, which pose a long-term threat for the environment (Suman et al., 2018). According to their role in biological systems, heavy metals can be grouped as essential and non-essential. Essential heavy metals such as Cu, Fe, Mn, Ni, and Zn are required for physiological and biochemical processes during plant life cycle (Cempel and Nikel, 2006); however, they may become toxic when present in excess. Non-essential heavy metals like Pb, Cd, As, and Hg are highly toxic with no known function in plants (Fasani et al., 2018) and may cause environmental pollution and severely affect a variety of physiological and biochemical processes in crop plants and reduce agricultural productivity (Clemens, 2006). They can enter into the food chain through crops and accumulate in the human body through biomagnification, thus posing a great threat to human health (Sarwar et al., 2010; Rehman et al., 2017).

Hence, it is necessary to take remediation measures to prevent heavy metals from entering into terrestrial, atmospheric, and aquatic environments, and mitigate the contaminated land (Gerhardt et al., 2017; Hasan et al., 2019). So far, there are a variety of remediation approaches that have been developed to reclaim heavy metal-contaminated soil. These measures are mainly based on mechanical or physio-chemical techniques, such as soil incineration, excavation and landfill, soil washing, solidification, and electric field application (Sheoran et al., 2011; Wuana and Okieimen, 2011; DalCorso et al., 2019). However, there are limitations reported on these physicochemical approaches such as high cost, inefficiency when contaminants are present at low concentrations, irreversible changes to the physicochemical and biological properties of soils, which lead to the deterioration of the soil ecosystem, introduction of secondary pollutions (Ali et al., 2013; DalCorso et al., 2019). Therefore, there is a need to develop cost-effective, efficient, and environment-friendly remediation technologies to reclaim heavy metal-contaminated soil.

Phytoremediation is a plant-based approach, which involves the use of plants to extract and remove elemental pollutants or lower their bioavailability in soil (Berti and Cunningham, 2000). Plants have the abilities to absorb ionic compounds in the soil even at low concentrations through their root system. Plants extend their root system into the soil matrix and establish rhizosphere ecosystem to accumulate heavy metals and modulate their bioavailability, thereby reclaiming the polluted soil and stabilizing soil fertility (Ali et al., 2013; Jacob et al., 2018; DalCorso et al., 2019). There are advantages of using phytoremediation, which include: (i) economically feasible—phytoremediation is an autotrophic system powered by solar energy, therefore, simple to manage, and the cost of installation and maintenance is low, (ii) environment and eco-friendly—it can reduce exposure of the pollutants to the environment and ecosystem, (iii) applicability—it can be applied over a large-scale field and can easily be disposed, (iv) it prevents erosion and metal leaching through stabilizing heavy metals, reducing the risk of spreading of contaminants, (v) it can also improve soil fertility by releasing various organic matters to the soil (Aken et al., 2009; Wuana and Okieimen, 2011; Jacob et al., 2018). During the past decades, numerous studies have been conducted to understand the molecular mechanisms underlying heavy metal tolerance and to develop techniques to improve phytoremediation efficiency. In the current review, the mechanisms of how heavy metals are taken up and translocated in plants are described, and the detoxification strategies (avoidance and tolerance) adopted by plants in response to heavy metal have been discussed. The main objective is to overview the recent advances in developing phytoremediation techniques, including the strategies to improve heavy metal bioavailability, tolerance, and accumulation. This review also highlights the application of genetic engineering to improve plant performance during phytoremediation.

## Uptake and Translocation of Heavy Metals in Plants

There are series of processes involved in accumulation of heavy metal in plants, including heavy metal mobilization, root uptake, xylem loading, root-to-shoot transport, cellular compartmentation, and sequestration. Heavy metal mostly exists as insoluble form in soil, which is not bioavailable to plants. Plants can increase their bioavailability by releasing a variety of root exudates, which can change rhizosphere pH and increase heavy metal solubility (Dalvi and Bhalerao, 2013). The bioavailable metal is sorbed at the root surface and moves across the cellular membrane into the root cells. The uptake of heavy metals into roots occurs mainly through two pathways, apoplastic pathway (passive diffusion) and symplastic pathway (active transport against electrochemical potential gradients and concentration across the plasma membrane). The common uptake of heavy metals via symplastic pathway is an energy-dependent process mediated by metal ion carriers or complexing agents (Peer et al., 2005).

After entering into root cells, heavy metal ions can form complexes with various chelators, such as organic acids. These formed complexes including carbonate, sulfate, and phosphate precipitate, are then immobilized in the extracellular space (apoplastic cellular walls) or intracellular spaces (symplastic compartments, such as vacuoles) (Ali et al., 2013). The metal ions sequestered inside the vacuoles may transport into the stele and enter into the xylem stream via the root symplasm (Thakur et al., 2016) and subsequently are translocated to the shoots through xylem vessels. Through apoplast or symplast, they are transported and distributed in leaves, where the ions are sequestered in extracellular compartments (cell walls) or plant vacuole, thereby preventing accumulation of free metal ions in cytosol (Tong et al., 2004).

### Heavy Metal Ion Transporter

Uptake and translocation of heavy metal in plant is mediated by a variety of molecules, including metal ion transporters and complexing agents. These specialized transporters (channel proteins) or H^+^-coupled carrier proteins are located in the plasma membrane of the root cell and are essential for the uptake of heavy metal ions from soil. They can transport specific metals across cellular membranes and mediate influx–efflux of metal translocation from roots to shoots (DalCorso et al., 2019). According to sequence homology, metal transporters identified, so far, have been classified into several families, such as ZIP, HMAs, MTPs, and NRAMPs.

Transporters of the ZIP family (ZRT–IRT-like proteins) are involved in heavy metal accumulation processes including uptake and transport of many cations (e.g., Fe, Mn, and Zn) from root to shoot (Guerinot, 2000). For example, Zn hyperaccumulator *Thlaspi caerulescens* and *Arabidopsis halleri* roots have enhanced Zn uptake in comparison to non-hyperaccumulator species, which is correlated with enhanced expression of some ZIP family members in hyperaccumulator (Assunção et al., 2001). The P1B-type ATPases of heavy metal transporting ATPases (HMAs) transporter family are involved in the transport of heavy metals (such as Zn, Cd, Co, and Pb) and play a vital role in metal homeostasis and tolerance (Axelsen and Palmgren, 2001; Williams and Mills, 2005). HMA3, a vacuolar P1B-ATPase, is involved in compartmentation of Zn, Cd, Co, and Pb by regulating their sequestration into the vacuole (Williams and Mills, 2005; Hanikenne and Baurain, 2014). Another transporter of the family, HMA4, is involved in long-distance root-to-shoot translocation of Zn and Cd (Verret et al., 2004). Overexpression of HMA4 enhanced Cd and Zn efflux from the root symplasm into the xylem vessels and promoted metal tolerance. Another group of transporters that tightly regulate metal homeostasis is metal transporter proteins (MTPs) family, which is involved in the translocation of metals (such as Zn and Ni) toward internal compartments and extracellular space (Gustin et al., 2011). MTP1, a vacuolar Zn^2+^/H^+^ antiporter, which localized at both vacuolar and plasma membrane, is involved in Zn accumulation as well as Zn tolerance (Desbrosses-Fonrouge et al., 2005). MTP members are also involved in Ni vacuolar storage in *Thlaspi goesingense* (Persans et al., 2001). The naturally resistant associated macrophage proteins (NRAMPs) are also involved in the transport of many heavy metal ions including Cu^2+^, Mn^2+^, Co^2+^, Fe^2+^, and Cd^2+^ (Supek et al., 1997; Cailliatte et al., 2010; Bastow et al., 2018). AtNRAMP1 is localized in the plasma membrane and mediates Fe and Mn transport (Cailliatte et al., 2010). NRAMP3 and NRAMP4 are localized in the tonoplast and mediate the export of stored Fe from the vacuole in germinating seed (Bastow et al., 2018).

Besides metal ion transporter, complexing agents including organic acids and amino acids act as metal ligands to mediate chelation of heavy metal ions. For example, citrate is a major chelator for Fe and Ni in the xylem (Tiffin, 1970; Lee et al., 1977), while Ni may also be chelated by histidine (Krämer et al., 1996).

## Detoxification Mechanism

Heavy metal detoxification is a key prerequisite for the implementation of phytoremediation (Thakur et al., 2016). Generally, there are two defense strategies adopted by plants to cope with the toxicity of heavy metals: avoidance and tolerance. By these two mechanisms, plants manage to maintain the cellular concentrations of heavy metals below the toxicity threshold levels (Hall, 2002).

### Avoidance

Avoidance strategy refers to the ability of plants to limit the uptake of heavy metals and restrict their movement into plant tissues through root cells (Dalvi and Bhalerao, 2013). It works as the first line of defense at extracellular level through a range of mechanisms such as root sorption, metal ion precipitation, and metal exclusion (Dalvi and Bhalerao, 2013). Upon exposure to heavy metals, plants first try to immobilize them either through root sorption or by modifying metal ions. A variety of root exudates, such as organic acids and amino acids, act as a heavy metal ligand to form stable heavy metal complexes in the rhizosphere (Dalvi and Bhalerao, 2013). Some root exudates can change the pH of rhizosphere, which lead to precipitation of heavy metals, thereby limiting their bioavailability and lessening the toxicity (Dalvi and Bhalerao, 2013). Through metal exclusion mechanism, exclusion barriers exist between the root system and the shoot system to limit the access of heavy metals from soil only to roots; the uptake and root-to-shoot transport is restricted to protect aerial parts against harmful heavy metals. Moreover, arbuscular mycorrhizas can restrict the entry of heavy metals into the root by absorption, adsorption, or chelation of heavy metals in the rhizosphere, thus, working as an exclusion barrier for heavy metal uptake (Hall, 2002). Embedding the heavy metals in the plant cell walls is another mechanism of heavy metal avoidance (Memon and Schröder, 2009). Cell wall pectins consist of carboxylic groups of polygalacturonic acids, which are negatively charged and able to bind heavy metals. Therefore, cell wall acts as a cation exchanger to restrict entry of free heavy metal ions into the cells (Ernst et al., 1992).

### Tolerance

Once the heavy metal ions get entry into the cytosol, tolerance strategy is adopted by the plants to cope with the toxicity of accumulated metal ions. It is the second line of defense at intracellular level through various mechanisms such as inactivation, chelation, and compartmentalization of heavy metal ions (Dalvi and Bhalerao, 2013).

When excess heavy metal ions are accumulated inside the cytosol, plants have to detoxify them in order to minimize their toxic effects (Manara, 2012). This is mainly achieved through chelation by complexation of heavy metal ions with ligands. Through chelation, the concentrations of free metal ions are reduced to relatively low levels. There are many organic and inorganic ligands in the cytoplasm that mediate heavy metal chelation. The organic compounds involved in heavy metal ion chelation include organic acids, amino acids, phytochelatins (PCs), metallothioneins (MTs), and cell wall proteins/pectins/polyphenols (Hall, 2002; Sharma and Dietz, 2006; Gupta et al., 2013b). Organic acids within cells prevent the persistence of heavy metals as free ions in the cytoplasm by complexing and reducing their bioavailability to plants. For example, citrate mediates the chelation of Ni in *T. goesingense* leaves (Krämer et al., 2000), while acetic and citric acids bind Cd in leaves of *Solanum nigrum* (Sun et al., 2006). In addition, malate is involved in chelation of Zn in *A. halleri* (Sarret et al., 2002). Heavy metal stress induces the accumulation of certain kinds of amino acid. For example, Cd can induce the production of cysteine in *Arabidopsis thaliana* (Domínguez−Solís et al., 2004), while Ni hyperaccumulation induces histidine accumulation (Harper et al., 1999). High accumulation of proline is also induced by Cd, Pb, Zn, and Cu stress (Roy and Bera, 2002). These amino acids can detoxify heavy metals by chelating heavy metal ions within cells and xylem sap (Rai, 2002). PCs and MTs are also induced in response to high levels of heavy metals. For example, Cd is chelated by PCs in tobacco leaves (Vögeli-Lange and Wagner, 1990), while MTs mediate the response to Cu stress in *Silene vulgaris*, as increased expression of MT gene is associated with enhanced Cu tolerance (van Hoof et al., 2001).

**FIGURE 1:**
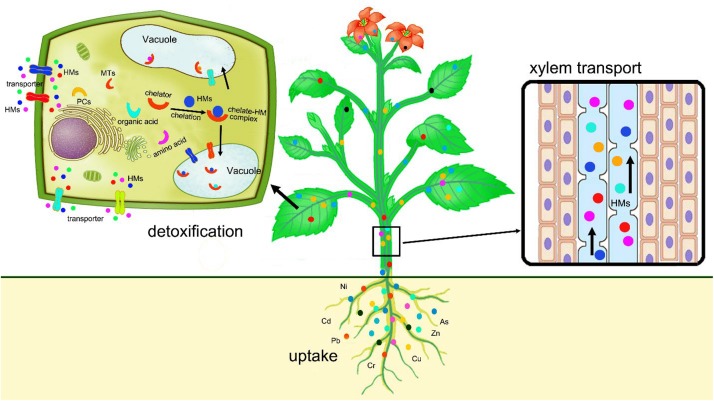
Schematic diagram shows the uptake, translocation, and sequestration of heavy metals in plants.

After chelation, the complexes of ligands with heavy metals are actively transported from the cytosol into inactive compartments, such as vacuole where the complexes are stored without toxicity (Tong et al., 2004). Sequestration and vacuolar compartmentalization provide an effective protection against the detrimental effects of heavy metals by removing toxic heavy metal ions from sensitive sites of the cell where cell division and respiration occur, thereby reducing the interactions between heavy metal ions and cellular metabolic processes and avoiding damages to cell functions (Sheoran et al., 2011). The uptake, translocation, and detoxification of heavy metals in plants are illustrated in Figure 1.

Besides vacuoles, heavy metal ions can be sequestrated and compartmentalized into other locations, such as leaf petioles, leaf sheathes, and trichomes (Robinson et al., 2003; Eapen and D’souza, 2005), where heavy metals cause less damage to the plant. Heavy metals can also be translocated to old leaves and removed from the plant body by natural leaf shedding (Thakur et al., 2016). For example, Zn is moved to *Plantago lanceolata* leaves just during the last week prior to leaf shedding and eventually removed from the plant after leaf fall (Ernst et al., 1992).

When the environment accumulates high levels of heavy metals, once the above-mentioned strategies are inadequate to detoxify the detrimental effects of heavy metals, the increased accumulation of metal ions in the cytoplasm trigger the production of reactive oxygen species (ROS). The excess production of ROS results in oxidative stress, which may cause disruption of cell homeostasis, inhibition of cellular processes, DNA damage, and protein oxidation (Huang et al., 2012; DalCorso et al., 2019). To cope with heavy metal-induced oxidative damage, plant cells activate the ROS-scavenging machinery by inducing antioxidant enzymes, such as superoxide dismutase (SOD), catalase (CAT), peroxidase (POD), and glutathione reductase (GR), as well as non-enzymatic antioxidant compounds including glutathione, flavonoids, carotenoids, ascorbate, and tocopherols (Gupta et al., 2009; Jozefczak et al., 2012; DalCorso et al., 2019). Hence, this anti-oxidative defense system of plants plays an important role in response to heavy metal stress.

## Phytoremediation

There are a number of phytoremediation strategies that are applicable for the remediation of heavy metal-contaminated soils, including (i) phytostabilization—using plants to reduce heavy metal bioavailability in soil, (ii) phytoextraction*—*using plants to extract and remove heavy metals from soil, (iii) phytovolatilization—using plants to absorb heavy metal from soil and release into the atmosphere as volatile compounds, and (iv) phytofiltration—using hydroponically cultured plants to absorb or adsorb heavy metal ions from groundwater and aqueous waste (Salt et al., 1995; Ernst, 2005; Marques et al., 2009). Other phytoremediation strategies include phytodegradation and rhizodegradation, which are used for breakdown of organic pollutants. Here, we focus on the most widely used phytoremediation strategies, phytostabilization, phytoextraction, phytovolatilization, and phytofiltration in the remediation of heavy metal-polluted soil.

### Phytostabilization

Phytostabilization is the use of metal-tolerant plant species to immobilize heavy metals belowground and decrease their bioavailability, thereby preventing their migration into the ecosystem and reducing the likelihood of metals entering into the food chain (Wong, 2003; Marques et al., 2009). Phytostabilization can occur through precipitation of heavy metals or reduction in metal valence in the rhizosphere, absorption, and sequestration within root tissues, or adsorption onto root cell walls (Ginn et al., 2008; Kumpiene et al., 2012; Gerhardt et al., 2017). Plant growth facilitates the preservation of soil health at heavy metal-polluted areas. The established vegetation cover cannot only stabilize heavy metals underground and minimize their leaching to groundwater but also prevents the dispersion of heavy metal-containing soil particles by wind (Vangronsveld et al., 2009; Mench et al., 2010). One of the advantages of phytostabilization is that disposal of hazardous biomass is not required when compared with phytoextraction (Wuana and Okieimen, 2011).

The selection of appropriate plant species is crucial for phytostabilization. To fulfill the requirement of highly effective phytostabilization, plants should be tolerant to the heavy metal conditions. As plant roots play a pivotal role to immobilize heavy metals, stabilize soil structure, and prevent soil erosion, plants should have dense rooting systems. Plants should be able to produce a large amount of biomass and grow fast to timely establish a vegetation cover in a specific site. In addition, the plant cover should be easy to maintain under field conditions (Berti and Cunningham, 2000; Marques et al., 2009). Many plant species, which meet the above requirements, have been identified and used for phytostabilization of heavy metal-polluted soils (for a comprehensive review, see Burges et al., 2018).

To improve phytostabilization efficiency, organic or inorganic amendments can be added to the contaminated soil. These soil amendments can alter metal speciation, reduce heavy metal solubility and bioavailability by changing pH value and redox status of the soil (Alvarenga et al., 2009; Epelde et al., 2009; Burges et al., 2018). Moreover, the application of amendments can increase the organic matter content and essential nutrients of the soil and improve physicochemical and biological properties, which can benefit plant colonization and improve water-holding capacity.

Interestingly, microorganisms living in the rhizosphere, such as bacteria and mycorrhiza, can assist phytostabilization. These microorganisms can improve efficiency of heavy metal immobilization through adsorbing metals onto their cell walls, producing chelators and promoting precipitation processes (Göhre and Paszkowski, 2006; Mastretta et al., 2009; Ma et al., 2011). They can also increase plant root surface and depth to facilitate phytostabilization and even serve as a filtration barrier against heavy metal ion translocation from roots to shoots (Göhre and Paszkowski, 2006).

### Phytoextraction

Phytoextraction is the use of plants to take up contaminants from soil or water, and translocate and accumulate those contaminants in their aboveground biomass (Salt et al., 1995; Jacob et al., 2018). In recent times, phytoextraction is the most important phytoremediation technique for reclamation of heavy metals and metalloids from the polluted soil (Ali et al., 2013; Sarwar et al., 2017). Unlike phytostabilization, by which plants only temporarily contain heavy metals, and these heavy metals still remain belowground, phytoextraction is a permanent solution for the removal of heavy metals from polluted soil. Therefore, it is more suitable for commercial application.

The process of phytoextraction of heavy metals includes a few steps: (i) mobilization of heavy metals in rhizosphere, (ii) uptake of heavy metals by plant roots, (iii) translocation of heavy metal ions from roots to aerial parts of plant, (iv) sequestration and compartmentation of heavy metal ions in plant tissues (Ali et al., 2013). The efficiency of phytoextraction relies on a few factors such as plant selection, plant performance, heavy metal bioavailability, soil, and rhizosphere properties. Therefore, the strategies to improve phytoextraction efficiency are developed in light of those aspects and are discussed below.

Appropriate selection of the plant species is vital for effective phytoextraction. The plant species for phytoextraction should possess the following characteristics: (i) high tolerance to the toxic effects of heavy metals, (ii) high extraction ability with accumulation of high levels of heavy metals in aboveground parts, (iii) fast growing with high biomass production, (iv) abundant shoots and extensive root system, (v) good adaptation to prevailing environment, strong ability to grow in poor soils, easy cultivation and harvest, (vi) highly resistant to pathogens and pests, be repulsive to herbivores to avoid heavy metals entering into the food chain (Seth, 2012; Ali et al., 2013).

Among these characteristics, metal-accumulating capacities and aboveground biomass are the key factors that determine the phytoextraction potential of a plant species. Therefore, two different strategies for plant selection are being employed: (i) the use of hyperaccumulator plants, which can accumulate heavy metals in aboveground parts to a greater extent and (ii) the use of plants with high aboveground biomass production, which may have lower metal-accumulating capacities, but overall accumulation of heavy metals is comparable to that of hyperaccumulators (Robinson et al., 1998; Salt et al., 1998; Ali et al., 2013).

Generally, hyperaccumulators are plant species capable of accumulating very high levels of heavy metals in their aboveground parts without phytotoxicity symptoms (Rascio and Navari-Izzo, 2011; van der Ent et al., 2013). The naturally occurring heavy metal hyperaccumulator can accumulate metals at levels 100-fold greater than common non-hyperaccumulating species under the same conditions (Rascio and Navari-Izzo, 2011). Strictly, the definition of hyperaccumulator should meet the following criteria: (1) the shoot-to-root ratio of heavy metal concentration is greater than 1, which is a sign of efficient ability to transport metals from roots to shoots (McGrath and Zhao, 2003; Marques et al., 2009); (2) the shoot-to-soil ratio of heavy metal concentration is greater than 1, indicating a higher capability to take up heavy metals from soil (McGrath and Zhao, 2003); and (3) the concentration of the metal in the shoot is higher than 10 mg/kg for Hg, 100 mg/kg for Cd and Se, 1,000 mg/kg for Co, Cu, Cr, Ni, and Pb, and 10,000 mg/kg for Zn and Mn (Baker and Brooks, 1989).

Searching for effective hyperaccumulators is a key and the most straightforward strategy for successful phytoremediation of heavy metals. Currently, more than 450 plant species from at least 45 angiosperm families have been identified as metal hyperaccumulators so far (Suman et al., 2018), ranging from annual herbs to perennial shrubs and trees, such as Brassicaceae, Fabaceae, Euphorbiaceae, Asterraceae, Lamiaceae, and Scrophulariaceae families (Salt et al., 1998; Dushenkov, 2003). Some species can even accumulate more than two elements, such as *Sedum alfredii*, which can hyperaccumulate Zn, Pb, and Cd (He et al., 2002; Yang et al., 2002, 2004). A list of some plants, which show high capacity of heavy metal accumulation is given in Table 1. However, using edible crops for phytoremediation should be avoided as heavy metals can accumulate in edible parts of the plant and thus enter into the food chain by human or animal consumption, raising concerns on human health. Hence, selection of the non-edible hyperaccumulators is a key for efficient and safe phytoremediation of heavy metals.

**TABLE 1:** List of some plants tested for heavy metals accumulation.

Heavy metal	Plant species	Maximum concentration in plant (mg/kg)	References
As	*Pteris vittata*	8331	Kalve et al., 2011
	*Pteris ryukyuensis*	3647	Srivastava et al., 2006
	*Pteris quadriaurita*	2900	Srivastava et al., 2006
	*Corrigiola telephiifolia*	2110	García-Salgado et al., 2012
	*Pteris biaurita*	2000	Srivastava et al., 2006
	*Pteris cretica*	1800	Srivastava et al., 2006
	*Eleocharis acicularis*	1470	Sakakibara et al., 2011

Cd	*Phytolacca Americana*	10,700	Peng et al., 2008
	*Sedum alfredii*	9000	Xiong et al., 2004
	*Prosopis laevigata*	8176	Buendía-González et al., 2010
	*Arabis gemmifera*	5600	Kubota and Takenaka, 2003
	*Salsola kali*	2075	de la Rosa et al., 2004
	*Thlaspi caerulescens*	1140	Brown et al., 1994
	*Azolla pinnata*	740	Rai, 2008
	*Deschampsia cespitosa*	236.2	Kucharski et al., 2005
	*turnip landraces*	52.94–146.95	Li et al., 2016

Co	*Haumaniastrum robertii*	10,232	Marques et al., 2009

Cr	*Pteris vittata*	20,675	Kalve et al., 2011

Cu	*Eleocharis acicularis*	20,200	Sakakibara et al., 2011
	*Aeolanthus biformifolius*	13,700	Chaney et al., 2010
	*Ipomoea alpine*	12,300	Mitch, 2002
	*Haumaniastrum katangense*	8356	Sheoran et al., 2009
	*Pteris vittata*	91.975	Wang et al., 2012

Hg	*Achillea millefolium*	18.275	Wang et al., 2012
	*Marrubium vulgare*	13.8	Rodriguez et al., 2003
	*Rumex induratus*	6.45	Rodriguez et al., 2003
	*Silene vulgaris*	4.25	Pérez-Sanz et al., 2012
	*Festuca rubra*	3.17	Rodriguez et al., 2003
	*Poa pratensis*	2.74	Sas-Nowosielska et al., 2008
	*Hordeum* spp	2.35	Rodriguez et al., 2003
	*Helianthus tuberosus*	1.89	Sas-Nowosielska et al., 2008
	*Armoracia lapathifolia*	0.97	Sas-Nowosielska et al., 2008
	*Juncus maritimus*	0.315	Zheng et al., 2011
	*Cicer arietinum*	0.2	Wang et al., 2012

Mn	*Schima superba*	62412.3	Yang et al., 2008
	*Macadamia neurophylla*	51,800	Sheoran et al., 2009
	*Maytenus bureaviana*	33750	Marques et al., 2009
	*Alyxia rubricaulis*	14000	Marques et al., 2009; Chaney et al., 2010

Ni	*Psychotria douarrei*	47,500	Cunningham and Ow, 1996
	*Phyllanthus serpentinus*	38,100	Chaney et al., 2010
	*Alyssum murale*	4730–20,100	Bani et al., 2010
	*Alyssum markgrafii*	19,100	Bani et al., 2010
	*Alyssum corsicum*	18,100	Li et al., 2003
	*Berkheya coddii*	18,000	Mesjasz-Przybyłowicz et al., 2004
	*Alyssum pterocarpum*	13,500	Li et al., 2003
	*Alyssum caricum*	12,500	Li et al., 2003
	*Alyssum heldreichii*	11,800	Bani et al., 2010
	*Alyssum bertolonii*	10,900	Li et al., 2003
	*Alyssum serpyllifolium*	10,000	Prasad, 2005
	*Isatis pinnatiloba*	1441	Altinözlü et al., 2012

Pb	*Medicago sativa*	43,300	Koptsik, 2014
	*Brassica juncea*	10,300	Koptsik, 2014
	*Brassica nigra*	9400	Koptsik, 2014
	*Thlaspi rotundifolium*	8200	Cunningham and Ow, 1996
	*Helianthus annuus*	5600	Koptsik, 2014
	*Euphorbia cheiradenia*	1138	Chehregani and Malayeri, 2007
	*Betula occidentalis*	1000	Koptsik, 2014
	*Deschampsia cespitosa*	966.5	Kucharski et al., 2005

Se	*Lecythis ollaria*	18,200	Marques et al., 2009
	*Astragalus racemosus*	14,920	Marques et al., 2009

Zn	*Thlaspi caerulescens*	51,600	Cunningham and Ow, 1996
	*Eleocharis acicularis*	11,200	Sakakibara et al., 2011
	*Thlaspi calaminare*	10,000	Sheoran et al., 2009
	*Deschampsia cespitosa*	3614	Kucharski et al., 2005

Although many hyperaccumulators have been identified and used in phytoremediation of heavy metals, most of them are short-lived with low biomass production and slow growth rate, which limit the efficiency of phytoextraction. Alternatively, high biomass producing non-hyperaccumulators can be used for phytoextraction of heavy metals. Although they usually accumulate lower concentrations of heavy metals in their aboveground tissues on a per mass basis, the high biomass production can compensate for the lower phytoextraction efficiency, and the overall accumulation levels may even be higher than that of hyperaccumulators (Ebbs et al., 1997; Vangronsveld et al., 2009; Vamerali et al., 2010).

High biomass producing crops, such as *Helianthus annuus*, *Cannabis sativa*, *Nicotiana tabacum*, and *Zea mays*, have been reported to effectively remove heavy metals from contaminated soil through phytoextraction (Kayser et al., 2000; Tlustoš et al., 2006; Vangronsveld et al., 2009; Herzig et al., 2014). Grasses can also be used for phytoextraction because of their short life cycle, high growth rate, more biomass production, and high tolerance to abiotic stresses (Malik et al., 2010). For example, *Trifolium alexandrinum* is selected as a suitable candidate for phytoextraction of Cd, Pb, Cu, and Zn, owning to its fast growth, resistance to pollution loads, high biomass, and multiple harvests in a single growth period (Ali et al., 2012). Woody species, like trees, are used for phytoextraction due to several advantages. Woody species can produce a very high amount of biomass when compared to herbs and shrubs, which facilitate the accumulation of high levels of heavy metals in their aboveground biomass. They have a deep root system, which can effectively reduce soil erosion and prevent the dispersal of contaminated soil to the surrounding environment (Suman et al., 2018). In addition, trees are preferred than crop plants for phytoremediation due to their non-edible characteristics, which means there is a lower probability of the heavy metals entering into the food chain via trees (Burges et al., 2018).

### Phytovolatilization

Phytovolatilization is a phytoremediation strategy using plants to take up pollutants from soil, convert these toxic elements into less toxic volatile form, and subsequently release them into the atmosphere by plant transpiration process via the leaves or foliage system. This approach can be applied for detoxification of organic pollutants and some heavy metals like Se, Hg, and As (Mahar et al., 2016). For example, members of the Brassicaceae family are good volatilizers of Se, such as *Brassica juncea* (Banuelos and Meek, 1990; Terry et al., 1992; Banuelos et al., 1993). Inorganic Se is first assimilated into the organic selenoamino acids selenocysteine (SeCys) and selenomethionine (SeMet). SeMet is biomethylated to form dimethylselenide (DMSe), which is volatile and can be dispersed into the air with less toxicity compared with inorganic Se (de Souza et al., 2000; Terry et al., 2000). Elemental form of Hg is liquid at room temperature and can be easily volatilized. Owing to its high reactivity, Hg exists mainly as a divalent cation Hg^2+^ after release into the environment (Marques et al., 2009). After taken up either by root or leaf absorption, methyl-Hg is converted to ionic Hg, which is later transformed into relatively less toxic elemental form and volatilized into the atmosphere (Bizily et al., 2000).

The advantage of phytovolatilization compared with other phytoremediation strategies is that heavy metal (metalloid) contaminants are removed from the site and dispersed as gaseous compounds, without any need for plant harvesting and disposal. However, as a remedial strategy, phytovolatilization does not remove the pollutants completely—the pollutants are still in the environment. It only transfers pollutants from soil to atmosphere, where the toxic volatile compounds will contaminate the ambient air. Moreover, they may be redeposited to the soil by precipitation (Vangronsveld et al., 2009). Thus, a risk assessment is required before its application in the field.

### Phytofiltration

Phytofiltration is the use of plant roots (rhizofiltration), shoots (caulofiltration), or seedlings (blastofiltration) to remove pollutants from contaminated surface waters or waste waters (Mesjasz-Przybyłowicz et al., 2004). During rhizofiltration, heavy metals are either adsorbed onto the root surface or absorbed by the roots. Root exudates can change rhizosphere pH, which leads to the precipitation of heavy metals on plant roots (Javed et al., 2019), further minimizing movement of heavy metals to underground water.

The plants used for rhizofiltration are hydroponically grown in clean water to develop a large root system first; then, the clean water is substituted with polluted water to acclimate the plants. After acclimation, the plants are transferred to the contaminated site for removal of heavy metals. Once the roots become saturated, they are harvested and disposed (Wuana and Okieimen, 2011). Ideally, plants used for rhizofiltration should have a dense root system, high biomass production, and be tolerant to heavy metal. Both terrestrial and aquatic plants can be used for rhizofiltration. For remediation of wetland water, aquatic species such as hyacinth, azolla, duckweed, cattail, and poplar are commonly used due to their high accumulation of heavy metals, high tolerance, or fast growth and high biomass production (Hooda, 2007). Terrestrial plants such as Indian mustard (*B. juncea*) and sunflower (*H. annuus*) have longer and hairy root system compared with aquatic plants. They also show good capacities to accumulate heavy metals during rhizofiltration (Tomé et al., 2008; Rezania et al., 2016; Dhanwal et al., 2017).

## Improving Plant Performance

The selected plant species with phytoremediation potential have few limitations, such as slow growing, which limit rapid and large-scale applications of these plants (Sarwar et al., 2017) and adaptation to a variety of environmental conditions like nutrient-poor soils (Gerhardt et al., 2017). Hence, to minimize these limitations, a strategy is developed through modifying and improving certain traits of these plants to ensure their ability for effective phytoremediation.

Traditional breeding (plant hybridization) or genetic engineering (creation of transgenic plants) are employed to either improve growth rate and biomass of hyperaccumulator or introduce hyperaccumulation traits to fast growth, high biomass plants (DalCorso et al., 2019). Brewer et al. (1999) used electrofusion to fuse protoplasts isolated from the Zn hyperaccumulator *T. caerulescens* and *Brassica napus*. The selected hybrids (somatic hybrid), which have enhanced hyperaccumulation capability and tolerance derived from *T. caerulescens* and higher biomass production derived from *B. napus* (Brewer et al., 1999), showed the ability to accumulate high levels of Zn and Cd. This study indicated that transfer of the metal hyperaccumulation trait to high biomass plants is feasible through somatic hybridization. Similarly, Nehnevajova et al. (2007) used chemical mutagen ethyl methanesulfonate (EMS) to treat sunflowers and obtained sunflower “giant mutant,” which exhibited a significantly enhanced heavy metal extraction ability with 7.5 times accumulation for Cd, 9.2 times for Zn, and 8.2 times for Pb compared to control plants (Nehnevajova et al., 2007).

### Genetic Engineering

Genetic engineering has been proved as a promising technique for improving phytoremediation abilities of plants toward heavy metal pollution. To genetically modify plants, a foreign source of gene from an organism, such as a plant species or even bacteria or animals, is transferred and inserted into the genome of a target plant. After DNA recombination, the foreign gene is inherited and confers specific traits to the plants. Compared to the traditional breeding, genetic engineering has the advantages to modify plants with desirable traits for phytoremediation in a much shorter time. Moreover, genetic engineering can even transfer desirable genes from hyperaccumulator to sexually incompatible plant species, which is impossible to achieve through traditional breeding methods such as crossing (Berken et al., 2002; Marques et al., 2009). Therefore, using genetic engineering to develop transgenic plants with the desired traits has shown attractive prospects in the field of phytoremediation. Technically, modifying fast-growing, high-biomass species to obtain high tolerance and high heavy metal accumulation ability is more applicable than engineering hyperaccumulators to get high-biomass production. Hence, in most applications, fast-growing, high-biomass plants are engineered either to enhance tolerance against heavy metals or to increase heavy metal-accumulation ability, which are the key properties of hyperaccumulators. Therefore, the selection of genes for genetic engineering should base on the knowledge of heavy metal tolerance and accumulation mechanisms in plants.

Heavy metals may cause excessive production of ROS and result in oxidative stress, so heavy metal tolerance is usually manifested by the strength of oxidative stress defense system. Therefore, the most common strategy to increase heavy metal tolerance is to enhance antioxidant activity (Koźmińska et al., 2018), which can be achieved by overexpression of genes involved in antioxidant machinery. To increase heavy metal accumulation through genetic engineering, the common strategy is to introduce and overexpress genes that are involved in the uptake, translocation, and sequestration of heavy metals (Mani and Kumar, 2014; Das et al., 2016). Hence, genes encoding heavy metal/metalloid transporters can be transferred and overexpressed in target plants to improve heavy metal accumulation. These genes encode metal ion transporters including ZIP, MTP, MATE, and HMA family members, which are discussed previously. As metal chelators act as metal-binding ligands to improve heavy metal bioavailability, promote heavy metal uptake and root-to-shoot translocation, as well as mediate intracellular sequestration of heavy metal ions in organelles, it is a promising strategy to increasing heavy metal accumulation by promoting the production of metal chelators via genetic engineering. By overexpression of genes encoding natural chelators, heavy metal uptake and translocation can be improved (Wu et al., 2010).

Although genetic engineering approach has shown attractive prospects on improving plant performance in phytoremediation of heavy metals, there are also a few setbacks that remain. As the mechanisms of detoxification and accumulation of heavy metals are very complicated and involve a number of genes, genetic manipulation of multiple genes to improve desired traits is time and effort consuming and usually not successful. Another issue is that genetically modified plants are difficult to gain approval for field testing in some areas of the world due to the risk raised on food and ecosystem safety. Therefore, alternative approaches are required to improve plant performance in phytoextraction once genetic engineering is impracticable.

### Using Microbes to Improve Plant Performance

Use of plant-associated microorganisms (rhizosperic microorganisms) is another approach to improve plant performance for phytoremediation. The microbial community of the rhizosphere may directly stimulate root proliferation and, thus, promote plant growth, increase heavy metal tolerance and plant fitness (Gupta et al., 2013a; Fasani et al., 2018).

It has been shown that plant growth-promoting rhizobacteria (PGPR) have large potential to improve phytoremediation efficiency. PGPR can promote plant growth and fitness, protect plants against pathogens, increase plant tolerance to heavy metals, improve plant nutrient uptake as well as heavy metal uptake, and translocation (Ma et al., 2011). This is achieved by producing various compounds, such as organic acids, siderophores, antibiotics, enzymes, and phytohormones (Ma et al., 2011). PGPR can synthesize the 1-aminocyclopropane-1-carboxylate (ACC) deaminase, which degrades the ethylene precursor ACC. Through producing ACC deaminase, PGPR is able to lower ethylene production, thus, promote plant growth (Arshad et al., 2007; Glick, 2014). Plants inoculated with PGPR containing ACC deaminase showed enhanced biomass production as manifested by extensive root and shoot densities, resulting in enhanced uptake of heavy metals and increased phytoremediation efficiency (Huang et al., 2004; Arshad et al., 2007). PGPR can also produce bacterial auxin (IAA) to stimulate lateral root initiation and root hair development, thus promoting plant growth and facilitating phytoremediation (Glick, 2010; DalCorso et al., 2019).

Arbuscular mycorrhizal fungi (AMF) is another important microbial community that can assist plants for phytoremediation. The presence of AMF in rhizospheres increases the absorptive surface area of plant roots through the extensive hyphal network, thus, enhancing water and nutrient uptake as well as heavy metal bioavailability (Göhre and Paszkowski, 2006). AMF can also produce phytohormones to promote plant growth and aid phytoremediation (Vamerali et al., 2010).

## Increasing Bioavailability of Heavy Metals

Beyond plant selection and performance, increasing heavy metal bioavailability is another important strategy to improve the efficiency of phytoextraction. The heavy metals present in the soil are not always readily available for bioaccumulation. Only a small portion of the total heavy metal content in the soil exist as soluble components in the soil and is ready for absorption by plants (Blaylock and Huang, 2000). Some heavy metals such as Zn and Cd are more mobile and bioavailable for plant than others (Lasat, 1999). According to the bioavailability of heavy metals/metalloids in the soil, heavy metals/metalloids can be classified as readily bioavailable heavy metals (Cd, Ni, Zn, As, Se, Cu), moderately bioavailable heavy metals (Co, Mn, Fe), and least bioavailable (Pb, Cr) (Prasad, 2003). Low bioavailability of certain heavy metals such as Pb seriously hinders the uptake of the metals from soil, thus reducing effective phytoextraction. The bioavailability of heavy metals in the soil is determined by their intrinsic solubility and soil properties, as well as the binding of heavy metals to soil particles. Various soil physicochemical factors, such as the presence of chelating agents, the soil pH, and microbial activity, have shown impacts on bioavailability and solubility of heavy metals in the soil (Rieuwerts et al., 1998; Wang et al., 2006).

A plant, itself, can employ various strategies to enhance heavy metal bioavailability. Root exudates acidify the rhizosphere by lowering soil pH, which promotes the desorption of heavy metals from insoluble complexes to form free ion, thus increasing the concentration of heavy metals in the soil (Thangavel and Subbhuraam, 2004). Plants can also secrete metal-mobilizing compounds in the rhizosphere, such as phytosiderophores, carboxylates, and organic acids, which affect physicochemical properties of the soil and facilitate heavy metal chelation, thereby increasing solubility, mobility, and bioavailability of heavy metals in the soil (Lone et al., 2008; Gerhardt et al., 2009; Robinson et al., 2009; Padmavathiamma and Li, 2012).

It has been reported elsewhere that microorganisms in the rhizosphere significantly increase heavy metal availability and uptake by plants (Vamerali et al., 2010; Sheoran et al., 2011). These microorganisms can secrete enzymes and chelate into the rhizosphere, which lead to the formation of heavy metal–chelate complexes, thus improving heavy metal uptake and translocation (Clemens et al., 2002). For example, PGPR and PGPE (plant growth-promoting endophytes) can increase solubility of water-insoluble Zn, Ni, and Cu through the secretion of protons or organic anions (Becerra-Castro et al., 2011). PGPR also secrete biosurfactants and siderophores to mobilize heavy metals in the soil. Siderophores are Fe chelators with strong affinity for ferric iron (Fe^3+^) and variable affinity for other heavy metals, such as Cd, Ni, As, and Pb (Schalk et al., 2011). Through chelation with these heavy metals, siderophores can enhance their bioavailability to both rhizobacteria and plants. In fact, using rhizobacteria to make heavy metal ions available has been proven effective. For example, Braud et al. (2009) inoculated siderophore-producing bacteria in an agricultural soil containing Cr and Pb with maize cultivated, and found that bioavailability of Cr and Pb was increased; their uptake by maize was also increased (Braud et al., 2009). In addition, mycorrhizal fungi can also change physicochemical properties of soil and chemical composition of plant root exudates, thereby affecting heavy metal bioavailability in the soil (Sarwar et al., 2017). For example, Chen et al. (2003) found that red clover (*Trifolium pratense* L.) inoculated with arbuscular mycorrhiza had a higher yield than uninoculated controls when grown in soil containing Zn. Further analysis indicated that mycorrhizal hyphae could directly absorb Zn from the soil and transfer it to the roots, thereby increasing its accumulation (Chen et al., 2003). Another widely considered strategy to increase heavy metal bioavailability is using chelating agents. When soil amendments containing chelating agents are added to the soil, the chelating agents form water-soluble heavy metal–chelate complexes with the heavy metals, which are more mobile and can be readily taken up by the plant (Wuana and Okieimen, 2011). Chelating agents can prevent precipitation and sorption of heavy metals in the soil and facilitate desorption of heavy metals from soil particles, thereby increasing heavy metal bioavailability (Salt et al., 1995; Ali et al., 2013). In practice, different chelating agents are used for the chelate-assisted phytoextraction, including synthetic and organic chelating agents. Synthetic chelating agents, such as ethylene diamine tetraeacetic acid (EDTA), ethylene glycol tetraeacitic acid (AGTA), and diethylene triamine pentaacetic acid (DTPA), can effectively increase heavy metal bioavailability and promote uptake by plants (Gupta et al., 2008; Sarwar et al., 2017). However, the poor biodegradability of these chelating agents results in their persistence in the soil, which raises great concern about metal leaching and detrimental effects on the environment (Smolińska and Król, 2012; Lee and Sung, 2014). As an alternative, organic chelating agents, such as citric acid, malic acid, acetic acid, and oxalic acid, have been proven to effectively form heavy metal complexes and enhance heavy metal bioavailability (Sarwar et al., 2017). These organic chelators have natural origins and are easily biodegradable in soil, which may introduce less risk to the environment than synthetic chelating agents (Souza et al., 2013); hence, it will be more promising to employ organic chelating agents for chelate-assisted phytoextraction.

**FIGURE 2:**
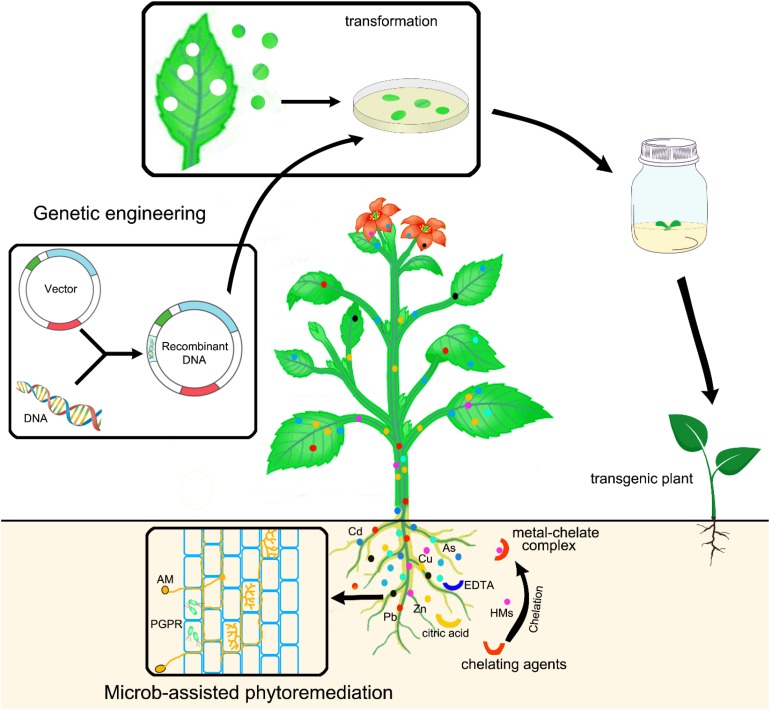
Schematic diagram illustrates strategies used to improve phytoremediation.

The strategies used to improve heavy metal phytoremediation, including genetic engineering, microbe-assisted, and chelate-assisted phytoremediation, are illustrated in Figure 2.

## Conclusion

Heavy metal pollution is a vital issue for agricultural production and food health due to the toxic effects and rapid accumulation in the environment. To prevent or mitigate heavy metal contamination and revegetate the contaminated soil, a variety of techniques have been developed. Phytoremediation has been proven to be a promising technique for revegetation of heavy metal-polluted soil with a good public acceptance and shows a variety of advantages compared with other physicochemical techniques. The application of heavy metal hyperaccumulators is the most straightforward approach for phytoremediation, and hundreds of hyperaccumulator plants have been identified so far. However, phytoremediation with these natural hyperaccumulators still suffers from a few limitations, as it is a time-consuming process, which takes a very long time to clean-up heavy metal-contaminated soil, particularly in moderately and highly contaminated sites. This may partially be due to slow growth rate and low biomass production of these hyperaccumulators. Therefore, improving plant performance is a critical step for developing high effective phytoremediation. Fortunately, genetic engineering approach has been emerging as a powerful tool to modify plants with desired traits such as fast grow, high biomass production, high heavy metal tolerance and accumulation, and good adaption to various climatic and geological conditions. Hence, good understanding of the mechanisms of heavy metal uptake, translocation, and detoxification in plants, and identification and characterization of different molecules and signaling pathway, will be of great importance for the design of ideal plant species for phytoremediation via genetic engineering. Genes involved in heavy metal uptake, translocation, sequestration, and tolerance can be manipulated to improve either heavy metal accumulation or tolerance in plants. In addition, chelating agents and microorganisms can be used either to increase heavy metal bioavailability, which facilitates heavy metal accumulation in plants, or to improve soil health and further promote plant growth and fitness.

Practically, single approach is neither possible nor sufficient for effective clean-up of heavy metal-polluted soil. The combination of different approaches, including genetic engineering, microbe-assisted and chelate-assisted approaches, is essential for highly effective and exhaustive phytoremediation in the future.

## Author Contributions

ZC conceived and initiated the project. AY, YW, and ZC contributed to plant science. ST contribute to the heavy metal chemistry. MM and SG contributed to soil science.

## Conflict of Interest

The authors declare that the research was conducted in the absence of any commercial or financial relationships that could be construed as a potential conflict of interest.
